# Dihydroartemisinin regulates immune cell heterogeneity by triggering a cascade reaction of CDK and MAPK phosphorylation

**DOI:** 10.1038/s41392-022-01028-5

**Published:** 2022-07-11

**Authors:** Qilong Li, Quan Yuan, Ning Jiang, Yiwei Zhang, Ziwei Su, Lei Lv, Xiaoyu Sang, Ran Chen, Ying Feng, Qijun Chen

**Affiliations:** 1 Key Laboratory of Livestock Infectious Diseases, Ministry of Education, Key Laboratory of Zoonosis, College of Animal Science and Veterinary Medicine, Shenyang Agricultural University, 120 Dongling Road, 110866 Shenyang, China; 2 Research Unit for Pathogenic Mechanisms of Zoonotic Parasites, Chinese Academy of Medical Sciences, 120 Dongling Road, 110866 Shenyang, China

**Keywords:** Immunotherapy, Molecular medicine

## Abstract

Artemisinin (ART) and dihydroartemisinin (DHA), apart from their profound anti-malaria effect, can also beneficially modulate the host immune system; however, the underlying molecular mechanisms remain unclear. Here, we report that DHA selectively induced T-cell activation, with an increased proportion of Ki67^+^CD4^+^ T cells, CD25^+^CD4^+^ T cells, interferon (IFN)-γ-producing CD8^+^ T cells, Brdu^+^ CD8^+^ T cells and neutrophils, which was found to enhance cellular immunity to experimental malaria and overcome immunosuppression in mice. We further revealed that DHA upregulated the expression of cell proliferation-associated proteins by promoting the phosphorylation of mitogen-activated protein kinase (MAPK), cyclin-dependent kinases (CDKs), and activator protein 1 in the spleen. This study is the first to provide robust evidence that DHA selectively induced the expansion of subsets of splenic T cells through phosphorylated CDKs and MAPK to enhance cellular immune responses under non-pathological or pathological conditions. The data significantly deepened our knowledge in the mechanism underlying DHA-mediated immunomodulation.

## Introduction

Dihydroartemisinin (DHA), an artemisinin (ART) derivative, exhibits more stable and antimalarial effect than ART;^1^ therefore, it has been widely used for the clinical treatment of malaria.^2^ Youyou Tu, a Nobel Prize Laureate owing to her discovery of ART, proposed that DHA could also be explored to treat other diseases, including lupus nephritis and lupus erythematosus.^2^ Accumulating evidence suggests that DHA, apart from its anti-parasitic potency, also possesses profound immune-regulatory properties.^3^ Recently, DHA was reported to be curative for colitis-associated colorectal cancer^4^ and psoriasis,^5^ which possibly functions in regulation of the T helper/T regulatory cell balance and inhibition of inflammation. DHA was also found to induce the expansion of splenic Interferon-γ (IFN-γ)^+^ CD8^+^ T cells and upregulated IFN-γ expression in tumor microenvironment.^6^ Additionally, DHA ameliorated inflammation through enhancing the suppressive function of regulatory T cells (Tregs).^7–9^ Although, the exact mechanism by which DHA beneficially regulated host immune system has not been well understood, it is postulated that DHA-induced mitogen-activated protein kinase (MAPK) activation might be involved.^10,11^

The three major MAPK families, including the classical MAPK (also known as ERK), C-Jun N-terminal kinase (JNK), and p38 kinase, constitute the signaling cascades that regulate cell division and differentiation.^12^ AP-1 family members are pivotal regulators of MAPK-dependent signaling cascades,^13^ which was known to participate in the regulation of cell proliferation and differentiation.^14^ Blocking AP-1 leads to a reduction in the expression of critical G1 cell cycle regulators and an increase in cyclin-dependent kinase (CDK) inhibition factors, resulting in reduced CDK2 and CDK4 activity, which causes a cell cycle arrest at the G1 phase.^15^

Eukaryotic cells are governed by a complex machinery that controls the pace of proliferation and orderly progression through the cycle phases G1, S, G2, and M,^16^ which are specifically regulated by CDK that binds cyclins.^17^ When CDK activity is low, the mini-chromosome maintenance (MCM) complex 2-7 (MCM 2-7), a substrate for CDKs, is loaded onto replication origins where it forms the pre-replicative complex;^18,19^ consequently, the cells start to differentiate from late M phase to the G1 phase. Upon initiation, MCM2-7 creeps with the replicating enzymes on DNA, thereby possibly acting as a replicative helicase to promote fork progression.^20^ Furthermore, CDKs can phosphorylate the retinoblastoma protein, which releases E2F, leading to increased expression of Ki67,^21^ a proliferation marker that promotes cell cycle progression and is also known as MKI67.

The aim of the present study was to further uncover the profound immune regulation property and decipher the underlying molecular mechanisms of DHA. The data demonstrated that DHA promoted splenic immune cell accumulation by enhancing MAPK-AP1-CDKs-Ki67-mediated cell cycle signaling, and increasing the expression of CDKs, MCMs, and Ki67 in the splenic CD4^+^ T cells. Thus, a previously unrecognized association between DHA- and MAPK/CDK-controlled cell proliferation has been established.

## Results

### DHA selectively promoted the proliferation of subgroups of splenic immune cells

To determine whether DHA regulates splenic cell proliferation and rearrangement, we administered DHA daily via gavage to healthy BALB/c mice. DHA administration was strongly and positively correlated with the spleen weight (*R* = 0.9621); however, it was not correlated with body weight (*R* = −0.1308, Supplementary Fig. 1a). In contrast, the administration of only CMC (the solvent control) was neither correlated with spleen weight (*R* = 0.0362) nor body weight (*R* = −0.0839, Supplementary Fig. 1b). The spleen length of the DHA-treated mice was also significantly increased (Supplementary Fig. 1c). Similar results were observed in male BALB/c mice and C57/BL6 mice.

**Fig. 1:**
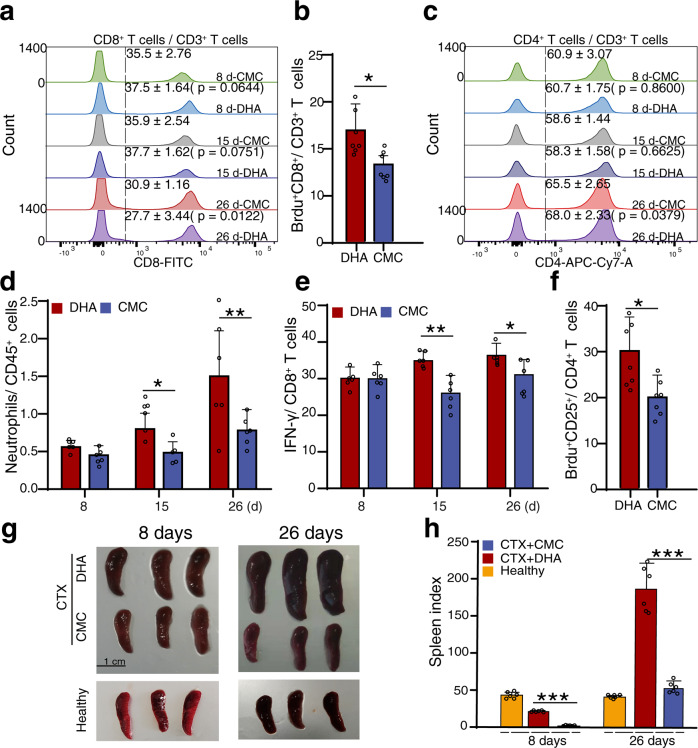
DHA promoted splenic cell proliferation and immune cell rearrangement in healthy and cyclophosphamide-induced immunosuppressed mice. **a** Representative flow cytometry histograms and percentages of positive cells from different DHA and CMC groups at different days after staining for cell surface CD45^+^CD3^+^CD8^+^. **b** Bar graphs represent significant increase in percentages of Brdu^+^ proliferative CD8 T cells in DHA-treated group compared to CMC-treated group on day 8 post treatment (*n* = 7). **c** Representative flow cytometry histograms and percentages of positive cells from different DHA and CMC groups at different days after staining for cell surface CD45^+^CD3^+^CD4^+^. **d**, **e** Bar graphs represent significant increases in percentages of Gr-1^+^ neutrophils (CD11b^+^ Gr-1^+^) or IFN-γ^+^ CD8 T cells of total CD45^+^ immune cells in DHA- and CMC-treated mice. **f** Bar graphs represent significant increases in percentages of Brdu^+^ CD25^+^ proliferative CD4 T cells in DHA-treated mice (*n* = 7). **g**, **h** Representative spleen images and quantitative analysis of spleen index of mice in cyclophosphamide-induced immunosuppressive mice and the healthy control mice. DHA=dihydroartemisinin group, CMC=CMC (carboxymethyl cellulose) solvent solution control. The results are representative of three independent experiments with a minimum of seven mice per group per experiment. *p < 0.05; **p < 0.01; ***p < 0.001

We recently explored the responses of immune cells to DHA treatment with a single-cell RNA sequencing approach, and observed that DHA selectively induced the expansion of IFNγ-producing CD8^+^ cytotoxic T cells, CD4^+^CD25^+^ T cells and neutrophils in the spleen.^10^ Here, it was found that, with prolonged DHA administration time, the proportion of total splenic CD8^+^ T cells increased initially but decreased later (Fig. 1a) compared to that in the control group. To further test whether DHA treatment would induce the proliferation of CD8^+^ T cells, we injected BrdU solution to label proliferating CD8^+^ T cells in vivo. The intensity of BrdU in the CD8^+^ T cells was also significantly increased (Fig. 1b), which aligned well with results showed in Fig. 1a. In addition, the proportion of total CD4^+^ T cells (Fig. 1c), neutrophils (Fig. 1d), and IFNγ-producing CD8^+^ cytotoxic T cells (Fig. 1e) was all increased with prolonged DHA administration compared to that of the control group. Moreover, a significant increase in CD4^+^CD25^+^ T cell proliferation in vivo represented by BrdU incorporation (Fig. 1f) under DHA treatment was in line with the results presented in Fig. 1b and our previous observation.^10^ Several previous studies reported that immunostimulators, such as TLR2 agonists and taurine, can increase spleen weight in CTX-immunosuppressed animals.^22,23^ Here, we also found that DHA treatment significantly reverted the CTX-induced spleen index reduction, whereas CMC did not show this effect (Fig. 1g, h).

### DHA activated the cell cycle signaling pathway

**Fig. 2:**
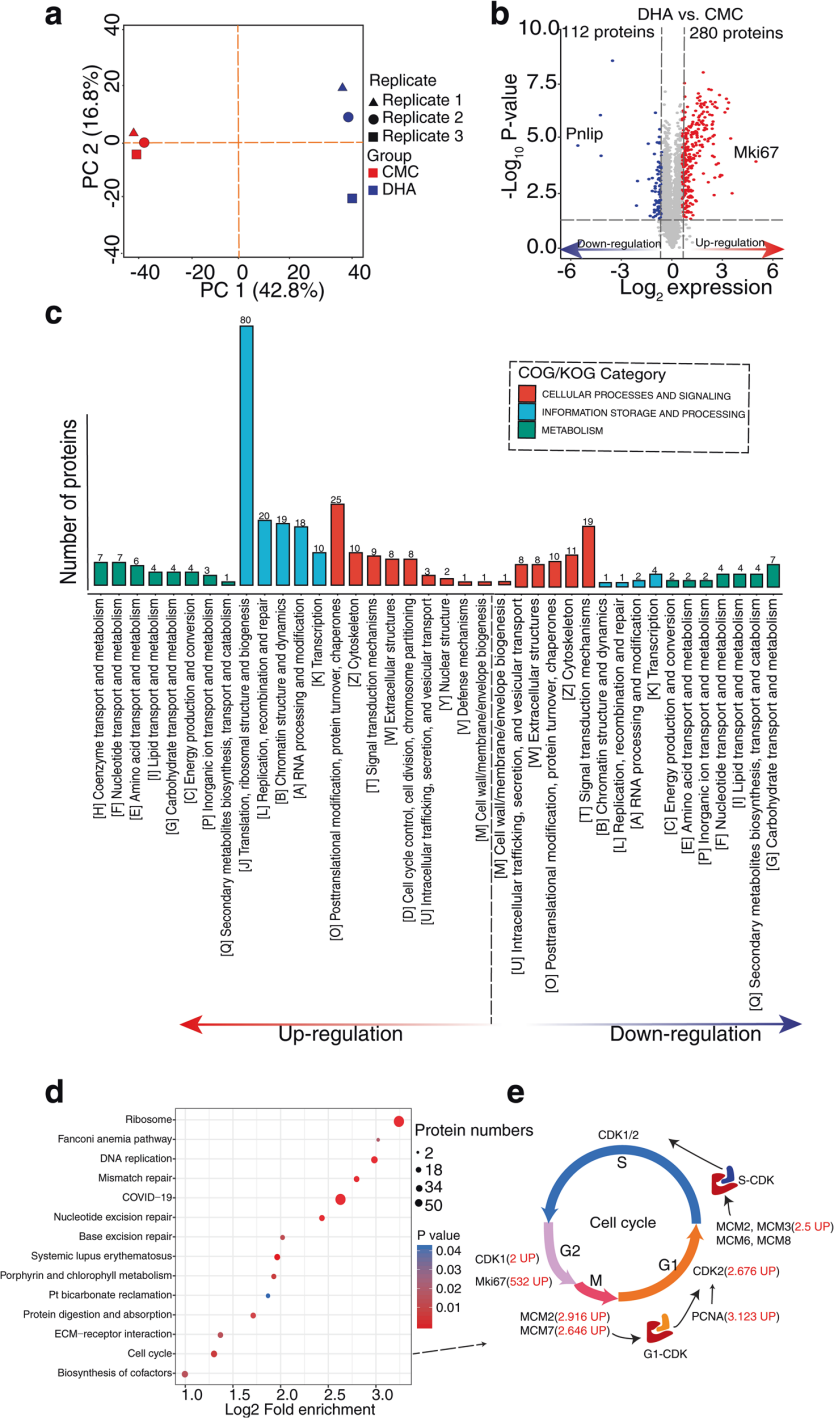
Quantitative proteomics revealed cell cycle progression through activation of cell cycle related proteins promoted by DHA. **a** The principal component analysis (PCA) at the proteomics level for a total of six spleen samples of the DHA- and CMC- gavage mice. **b** Differentially expressed proteins were displayed by volcano plots. The vertical lines correspond to 1.5-fold up and down, respectively, and the horizontal line represents the *P*-value cut-off of 0.05. Upregulation of protein expression in DHA group is shown in red, while downregulation is shown in blue. **c** KOG classifications of differentially expressed proteins (DEPs). The ordinate denotes the number of proteins in each KOG category. **d** A bubble chart showing the KEGG pathways. The bubble size represents the number of DEPs, and the bubble color represents the *p* value. **e** The pathway map showed the different key proteins involved in the cell cycle signaling pathways. DHA=dihydroartemisinin group, CMC=CMC (carboxymethyl cellulose) solvent solution control group

To further decipher the molecular mechanisms underlying the effects of DHA on splenic cell proliferation, we performed an unbiased quantitative proteomic comparison of splenic cells between DHA- and CMC-treated groups. Principal component analysis (PCA) showed that the three repeats of each sample clustered together, suggesting consistent reproducibility among the replicates (Fig. 2a). In total, 4143 proteins were quantified, of which 392 were differentially expressed between the DHA- and CMC-treated groups, including 280 upregulated and 112 downregulated proteins (|fold change| > 1.5 and *p* < 0.05, Fig. 2b and Data S1). Among them, Ki67, a major cell proliferation marker, exhibited the most significantly upregulated expression (log_2_ fold change = 9.06, *p* = 4.25E^−6^) in the DHA-treated group. In contrast, expression of the protease-sensitive pancreatic lipase (pnlip) was most significantly downregulated in the DHA-treated group (log_2_ fold change = −6.81, *p* = 0.001). Next, the KOG results showed that DHA markedly upregulated proteins with functions in translation, ribosomal structure and biogenesis, posttranslational modification, protein turnover, chaperone synthesis, replication, recombination, and repair pathways (Fig. 2c). The results of both KEGG and GSEA showed that proteins participating in ribosomal, DNA replication, and cell cycle pathways were upregulated (Fig. 2d and Supplementary Fig. 2).

The expression levels of key cell cycle regulators were analyzed to further understand the mechanism of DHA-induced splenic cell proliferation. The expression of key cell cycle regulatory proteins, including Ki67 (fold change = 532), proliferating cell nuclear antigen (PCNA, fold change = 3.123), MCM families (MCM2 fold change = 2.916, MCM3 fold change = 2.5, and MCM7 fold change = 2.646), CDK1 (fold change = 2), and CDK2 (fold change = 2.676), were upregulated by DHA (Fig. 2e). We further compared the expression of the key cell cycle regulatory proteins between DHA- and CMC-treated groups. The results of both Western blotting and immunohistochemical staining of the CDK1, CDK2, MCM2, and Ki67 were consistent with the findings of the proteomic analysis (Supplementary Fig. 3). In addition, seven differentially expressed proteins (DEPs) identified in the proteomic analysis were also validated by western blotting, and the results were also consistent with the quantitative proteomic results (Supplementary Fig. 3).

Further, more than 33% of DEPs were enriched as components of the ribonucleoprotein complexes (Supplementary Fig. 4). This suggest that DHA may be involved in the activation of cell cycle signaling pathway, since the acceleration of cell cycle is positively associated with an increased demand for ribosome synthesis.

### DHA treatment elevated the expression of Ki67 in subsets of splenic CD4^+^ T cells

**Fig. 3:**
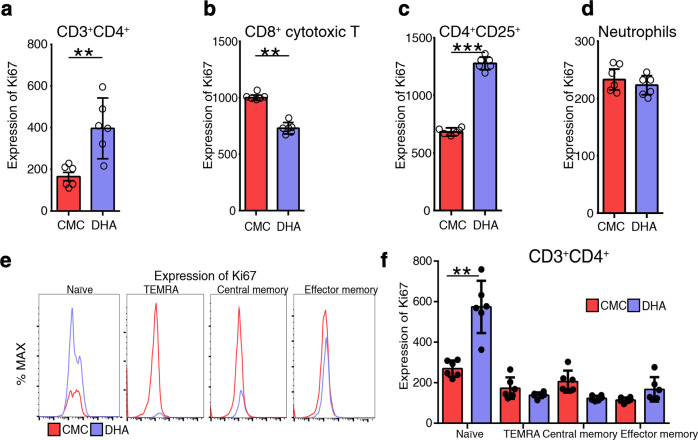
Assessment of Ki67 expression with flow cytometry. **a**–**d** Numbers indicate MFI of Ki67 expression at different subgroup in different groups. The expression of ki67 on the different subsets was calculated as MFI = positive staining (MFI)-isotype control (MFI). **e** Representative histograms showing differential expression of ki67 between DHA and CMC group in different CD3^+^CD4^+^ T cell subsets. **f** Representative histograms showing differential expression of ki67 between DHA and CMC group in different CD3^+^CD4^+^ T cell subsets (*n* = 6). Plot shows the increased expression of ki67 in the naïve CD4^+^ T cells (*n* = 6). A subset of effector memory T cells re-expresses CD45RA (termed TEMRA). DHA=dihydroartemisinin group, CMC=CMC (carboxymethyl cellulose) solvent solution control group; MFI=mean fluorescence intensities. *p < 0.05; **p < 0.01; ***p < 0.001

Since Ki67, a proliferative cell cycle marker, exhibited the most upregulated expression in response to DHA treatment identified in the above quantitative proteomic analysis, we further investigated the expression of Ki67 protein in different subsets of splenic immune cells of mice in either DHA-treated or the control group. Compared to that in the control group, the frequency of Ki67^+^ cells was significantly higher among CD3^+^CD4^+^ T cells, but not in the CD8^+^ T-cytotoxic cells in the DHA-treated group (Fig. 3a, b). Ki67 expression in CD4^+^CD25^+^ T cells also exhibited a significant increase in the DHA-treat group (Fig. 3c). In addition, Ki67 expression in neutrophils was slightly decreased, but not significantly different compared to that of the control group (Fig. 3d). Furthermore, the intracellular abundance of Ki67 protein was significantly upregulated in CD4 T_naïve_ cells after DHA treatment both in vivo (Fig. 3e, f) and in vitro (Supplementary Fig. 5a, b).

### DHA treatment significantly enhanced the kinase activity of MAPK/CDK pathway via protein phosphorylation

**Fig. 4:**
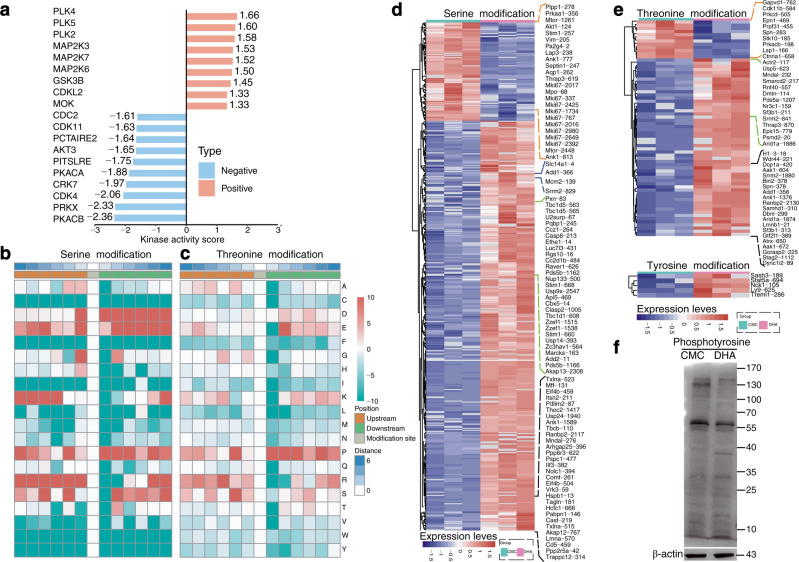
DHA-induced phosphorylation of MAPKs. **a** Analysis of the kinase activity between DHA to CMC group, where red indicates no inhibition (high kinase activity), blue indicates kinase inhibition (low kinase activity). **b**, **c** The motif enrichment heatmap of up-stream and down-stream amino acids of all identified modification sites. Red indicates that the amino acid is significantly enriched near the modification site and green significantly reduced near the modification site. **d**, **e** Heatmaps for visualizing are composed of the significant differentially modified sites between DHA and CMC group. **f** Western blot probed with a monoclonal anti-phosphotyrosine antibody to reveal the differences in protein phosphorylation between DHA-treated and untreated samples. DHA=dihydroartemisinin group, CMC=CMC (carboxymethyl cellulose) solvent solution control group

To further elucidate the regulatory mechanism of DHA on the expression of cell cycle related proteins, an unbiased phosphoproteomic analysis was performed. We firstly evaluated the kinase activity of the enzymes under DHA treatment compared to that from the control group. Based on differential kinase activity profiling, we found that DHA positively increased the kinase activity of CDK and MAPK (Fig. 4a). Proline, arginine, and serine residues in these kinases were phosphorylated at a high frequency downstream of p-serine and p-threonine. Aspartic acid and glutamic acid residues were phosphorylated at a high frequency downstream of p-serine, and lysine residues were phosphorylated at a high frequency upstream of p-serine (Fig. 4b, c). This suggests that CDK and MAPK might be the key kinases leading to the differential phosphorylation of cell cycle-related proteins induced by DHA. Furthermore, we found totally 485, 69, and 5 differentially regulated phospho-sites in serine, threonine, and tyrosine, respectively (Fig. 4d, e and Data S2). Of note, MCM2 and AKT1 have been recognized as critical components involved in the cell cycle signaling pathways. We found that MCM2 was activated via the phosphorylation of threonine 321 and serine 139. A previous study also reported that the phosphorylation of MCM2 by CDK promoted pre-replication complex assembly during cell cycle re-entry.^24^ As Akt1 is a negative regulator of CDK2, DHA induced a ~2-fold reduction in the relative phosphorylation level of AKT1 protein at serine -124. Finally, the phosphorylomes between DHA- and CMC-treated groups were confirmed with western blotting. DHA treatment resulted in significant changes in phosphorylation of the 130–170 kDa and ~40 kDa proteins (the molecular weight of MAPK and CDK is approximately 40 kDa) of the spleen tissues (Fig. 4f). These results indicated that DHA triggered MAPK or CDK signaling pathways by phosphorylation of cell cycle-related proteins.

### DHA promoted CDK and MAPK phosphorylation and c-Fos activation

**Fig. 5:**
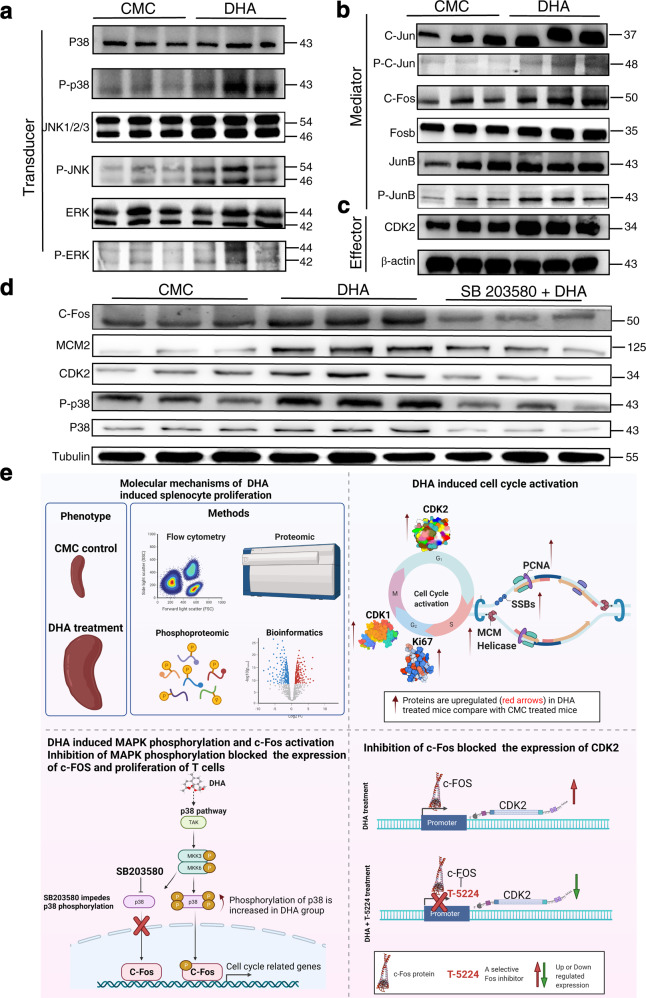
DHA regulated cell-cycle via the p38/JNK MAPK pathways. **a**–**c** Representative images of Western blot (WB) of phosphorylated MAPK proteins, AP-1 proteins, and CDK2. All experiments were performed in triplicate. Molecular weight (kDa) was labeled at the right. **d** The expression and phosphorylation of p38 and JNK from DHA-treated alone, the group with inhibitor and the control group were examined with specific antibodies. The expression of CDK2, MCM2, and c-Fos from both DHA-treated alone, the group with inhibitor and the control group were examined with specific antibodies. The effect of DHA was significantly inhibited by SB203580, a specific inhibitor of mitogen-activated protein kinase (p38), which downregulated the expression of c-Fos, MCM2, and CDK2. **e** Proposed mechanism of DHA-mediated regulation on p38 MAPK pathway and c-Fos complex. DHA induced an enlargement of the spleen and selectively promoted the proliferation of subgroups of splenic T cells. Importantly, DHA upregulated the expression of cell proliferation-associated proteins including CDK1, CDK2, Ssb, PCNA, MCMs, by promoting the phosphorylation of mitogen-activated protein kinase (MAPK) and the activation of c-Fos in the spleen. Inhibition of p38 MAPK and c-Fos blocked T cell proliferation. This figure was created using BioRender (https://biorender.com/). Agreement number is WJ23R5400C. DHA=dihydroartemisinin group, CMC=CMC (carboxymethyl cellulose) solvent solution control group. *N* = 3 (with biological triplicates) in the WB quantitation. p-p38, phosphorylated-p38 MAPK; p38, total p38 MAPK; SB203580 is a specific inhibitor of mitogen-activated protein kinase (p38); T-5224 is a specific inhibitor of C-Fos

The MAPK cascade consists of a relay of up to four kinases that phosphorylate and activate each other (usually to transmit signals from the cell surface to the nucleus), which controls the proliferation of immune cells.^25,26^ To further confirm that DHA could increase phosphorylation of the MAPK complex, the phosphorylation levels of p38 MAPK (p-p38 MAPK), JNK (p-JNK), and ERK (p-ERK) (relative to the total protein levels) were analyzed using Western blotting. Among the MAPK components, the phosphorylation levels of p38 and JNK, but not of ERK were most significantly upregulated in the DHA group (Fig. 5a), indicating that DHA increased MAPK phosphorylation.

P38 and JNK are well known to participate in activation of the AP-1 transcription factor, which promotes cell proliferation.^14^ Thus, to determine whether DHA promoted splenic cell proliferation via AP-1, we used Western blotting to compare AP-1 expression in the spleens of DHA-treated and control groups. C-Jun, phospho-c-Jun, c-Fos, and FosB, which are members of AP-1, and their expression was significantly upregulated in the DHA group compared to that in the CMC control group (Fig. 5b).

C-Jun and c-Fos have been shown to control the early G1 phase through CDK2 activation.^27,28^ Therefore, we compared CDK2 expression between the DHA- and CMC-treated groups and found a significant increase in CDK2 expression in the DHA group (Fig. 5c), and the result was consistent with the proteomic data (Data S1).

### Inhibition of p38 and c-Fos blocked T cell proliferation

To further verify the effect of DHA on the induction of MAPK pathway, a p38 inhibitor (SB203580) was tested in combination with DHA. The expression of c-Fos, MCM2, and CDK2 was reduced compared to that treated with DHA alone (Fig. 5d). SB203580 significantly reversed the effect of DHA on MAPK phosphorylation in the spleen. Importantly, SB203580 significantly inhibited the effect of DHA on Ki67 expression in splenic CD3^+^CD4^+^ T cells, CD4^+^ CD25^+^ T cells (Supplementary Fig. 6).

To further verify the effect of DHA on the c-Fos-CDK2 axis, a c-Fos inhibitor (T-5224) was applied in combination with DHA. Increased CDK2 expression was observed in mice treated with DHA compared to that of CMC-treated mice, whereas CDK2 expression was greatly reduced in mice treated with DHA in the presence of T-5224 (Supplementary Fig. 7a, b), implying that CDK2 expression was regulated by c-FOS. Additionally, in the immune-precipitation assay, no CDK was co-precipitated with p38, which suggest that there was no direct interaction between p38 and CDK2 (Supplementary Fig. 7c). Thus, it could be concluded that DHA enhanced splenocyte proliferation through the p38/MAPK- c-Fos axis (Fig. 5e). Based on this analysis, an atlas of DHA dynamically regulated proteins and pathways was constructed (Supplementary Fig. 8).

### Intermittent preventive treatment with DHA stimulated T cell activation and alleviated *Plasmodium* parasite infection

**Fig. 6:**
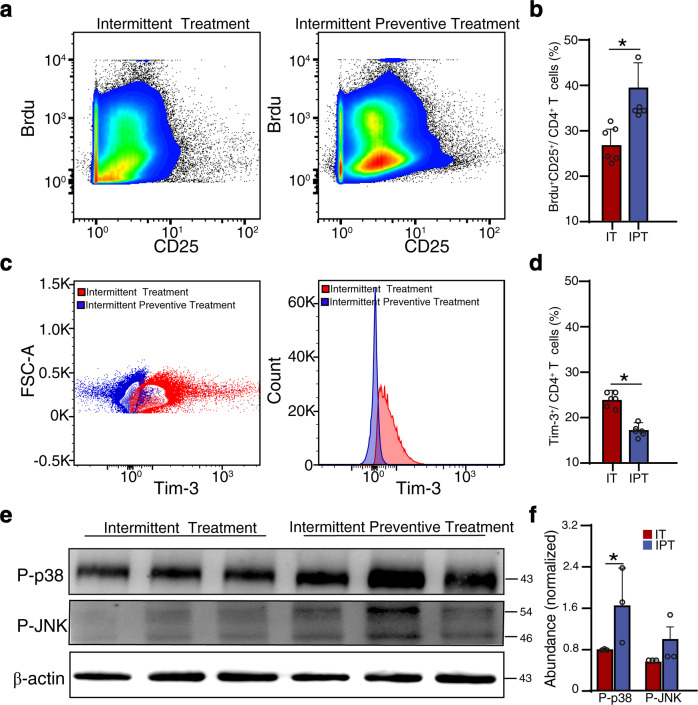
Intermittent preventive treatments with DHA-induced T cell activation. **a** Representative images of BrdU incorporation in CD25^+^ CD4^+^ T cells are shown. **b** More CD4^+^CD25^+^ T-lymphocyte proliferation was observed in the IPT group derived from the BrdU incorporation assays. **c** Histograms show representative flow cytometric stainings (left) and mean fluorescence intensities (right) of Tim-3, and **d** Bar graph shows differences in percentage of Tim-3^+^ (CD4-gated) T cells in the IT and IPT group. **e** Representative images of triplicate Western blot (WB) of phosphorylated- P38 and JNK, which were prominent in the IPT group than that in the IT group. Molecular weight (kDa) was labeled at the right. **f** Bar graph shows differences in normalized abundance of the two proteins between IT and IPT group. IT=Intermittent treatment, IPT=Intermittent preventive treatment. *N* = 3 (with biological triplicates) in WB quantitation. *p < 0.05. p-p38=phosphorylated-p38 MAPK

A DHA-piperaquine combination has been regarded as the best drug for intermittent preventive treatment of malaria due to their instant and long-lasting effects on malarial parasites.^29^ However, the immune-promoting effect of DHA may be an additional factor to the resistance to clinical malaria in the recipients. Therefore, we constructed a *Plasmodium*-infected mouse model subjected to intermittent treatment (IT) and intermittent preventive treatment (IPT) with DHA. IPT significantly delayed the onset of peak parasitemia compared to that with IT (*p* < 0.05, Supplementary Fig. 9). Previous studies indicated that CD25 was upregulated upon activation of T cells during malaria infection, which facilitated the clearance of *Plasmodium* parasite.^30–33^ In the IPT group, similar to that in healthy mice, T cells underwent significant activation (Fig. 6a) with elevated expression of the T cell activation marker CD25 as compared to that in the IT group (Fig. 6b). Furthermore, our previous studies showed that *Plasmodium* parasites could induce T cell exhaustion by promoting Tim-3 expression.^34,35^ In this study, we found that the expression of Tim-3 was significantly downregulated in the IPT group (Fig. 6c). Meanwhile, the proportion of Tim-3^+^CD4^+^ T cells in IPT group was lower than that in the IT group (Fig. 6d). We also noted that the IPT group exhibited significant upregulation of phosphorylated p38 (but not JNK) when compared with levels in the IT group (Fig. 6e, f). These findings indicate that DHA, apart from its potent parasiticide effect, could also indirectly alleviate malaria infection and revert CTX-induced immunosuppression.

## Discussion

The spleen is not only an important immune organ for the homing and differentiation of immune cells,^36^ but also functions as the site for filtration of apoptotic and infected cells such as malarial parasite-infected erythrocytes.^37^ DHA is a highly effective drug for the treatment of malaria^38,39^ and certain auto-immune diseases.^5^ In this study, we showed that DHA treatment selectively promoted the proliferation of a subpopulation of splenic immune cells (Fig. 1), which enhanced anti-malaria immunity. To further understand the underlying molecular mechanism, we generated the proteomic and phosphoproteomic profiles of immune cells after DHA treatment (Figs. 2–4). Our results further revealed that DHA activated the p38/MAPK-c-Fos signaling pathway (Fig. 5) to upregulate the expression of cell cycle-related genes (MCMs, Ki67, PCNA, and CDKs), thereby leading to the proliferation of splenic immune cells, which may facilitate the clearance of malarial parasite-infected erythrocytes (Fig. 6).

Although ART and its derivative DHA have long been proposed to regulate immunity against parasitic infections,^40^ the mechanism has not been well understood. In this study, DHA was administered to three models, including healthy, CTX-immunosuppressed, and malaria-infected mice (Figs. 1, 2, and 6). Similar results were observed in each of the three models independent of sex and mouse species, which revealed that DHA selectively induced the substantial proliferation of CD25^+^ CD4^+^ T cells and IFN-γ-producing CD8^+^ T cells in the spleen (Figs. 1 and 6). In addition, increased TNFα/IFN-γ has been positively correlated with T cell proliferation and parasite clearance.^41^ In this study, we directly demonstrated that DHA treatment promoted IFN-γ-producing CD8^+^ T cell proliferation (Fig. 1). Meanwhile, IPT significantly delayed the onset of peak parasitemia compared to that with IT (Supplementary Fig. 9). These results indicated that DHA could primarily enhance T cell activation and cellular immunity to parasite infection.

The spleen serves as a site of lymphocyte proliferation.^42^ In normal conditions, splenic lymphocyte proliferation is controlled by the MAPK-AP-1 signaling pathway.^43^ In this study, we identified several signaling pathways and proteins associated with cell proliferation that were activated or upregulated upon DHA treatment, including the ribosome, DNA replication, and cell cycle (Ki67, MCMs, PCNA, and CDKs). In addition, the expression of MAPKs (P38, JNK), their phosphorylated forms, and their downstream protein AP-1 were upregulated upon DHA treatment (Figs. 2–5). These findings suggest that DHA can activate the p38/MAPK-c-Fos signaling pathway in mice, resulting in lymphocyte proliferation in the spleen. In fact, elevated Ki67, MAPK, and AP-1 expression was not only observed early (at 8 days) upon DHA treatment but also in the late phase (at 26 days, data not shown).

CTX has been employed to treat a wide array of systemic autoimmune diseases.^44^ DHA, as a novel agent, has been exploited to treat experimental autoimmune diseases. Here, we provide strong evidence that DHA selectively enhances the proliferation of a subgroup of T cells in the spleen without immunosuppression. Our study might provide a foundation for the future exploration of CTX combined with DHA as a therapeutic agent for systemic autoimmune diseases.

In summary, this study provided strong evidence for the immune-regulatory mechanism of DHA, which beneficially enhanced host immunity to malaria infection by inducing the phosphorylation of critical proteins in the MAPK-AP-1 signaling pathway.

## Materials and methods

### Ethical statement

All procedures using the experimental animals were conducted in accordance with the animal husbandry guidelines of Shenyang Agricultural University. The institutional ethics committee of Shenyang Agricultural University approved the laboratory animal experiments (Permit No. SYXK < Liao>2017-0001).

### Mice

Female and male BALB/c mice with the N phenotype or C57/BL6 female mice (6–8-weeks-old, ~18–20 g) were purchased from the Liaoning Changsheng Biological Technology Company (Liaoning, China). Mice were housed under pathogen-free conditions and acclimatized for 1 week before experimentation. All experimental procedures performed on the experimental animals were conducted in accordance with the animal husbandry guidelines of Shenyang Agricultural University.

### Drug preparation

The 0.5% carboxymethyl cellulose (CMC) solution (Solarbio Company, Beijing, China, Catalog No. 9004-32-4) and a 0.1 mg/mL DHA solution (Puyi Biological Company, Nanjing, China, Catalog No. PY1835126Q, ~98% purity) were prepared as previously described.^3^ Briefly, 0.1 g of sodium CMC was dissolved in 20 mL of warm distilled water. When the solution was restored to room temperature, 0.2 g DHA was added and the solution was stirred for 1 h in dark. CMC without DHA was used as the solvent control. The SB 203580 and T-5224 are purchased from MedChemExpress company.

### Animal groups and treatment

Female BALB/c mice were randomized into six groups to receive DHA or CMC once per day for 8, 15, or 26 days. Data are representative of at least six mice per group. In the DHA treatment groups, each mouse was intragastrically administered 200 μL of 0.1 mg/mL DHA solution once per day. Mice in the CMC control groups were intragastrically administered 200 μL of a 0.5% CMC suspension once per day.

### In vivo inhibition experiments with SB203580 and T-5224

To further verify the effect of DHA on p38, a specific inhibitor SB203580 was administered intraperitoneally to the mice (5 mg/kg/day) for 8 days spontaneously treated with DHA (200 μL of 0.1 mg/mL). The control group included mice received DHA alone, CMC, and the dissolvent of SB203580 (DMSO in corn oil) with the dosage recommended by the manufacturer. To further verify the effect of DHA on c-FOS activity, T-5224, a specific inhibitor of c-Fos, was given to the mice with a dosage of 30 mg kg^−1^ bodyweight per day by oral gavage for 8 days together with DHA (200 μL, 0.1 mg/mL). The control group included mice received DHA alone, CMC, and an equal volume of PVP (vehicle for T-5224) with the dosage recommended by the manufacturer.

### Cyclophosphamide-induced immunosuppression

A cyclophosphamide (CTX) immunosuppressive model^45^ was used in this study. After 1 week of adaptation, female BALB/c mice were divided into five groups (*n* = 10 per group), including a blank control group. CMC control groups were administered treatments for 8 or 26 days (200 μL of 0.5% CMC each day), and DHA groups were administered drugs for 8 or 26 days (200 μL of 0.1 mg/mL each day). The blank group was administered equal volumes of saline. Before CMC or DHA administration, the mice in each group (except in the blank group) were intraperitoneally injected four times with 200 μL of CTX (5 mg/mL) every other day until day 7 to achieve complete immunosuppression.

### Intermittent preventive treatment with DHA to *Plasmodium berghei* infection

Each mouse was intraperitoneally (i.p.) injected with 10^3^ *P. berghei* ANKA-infected red blood cells. Parasitemia was examined from Giemsa-stained blood smears every 2 days post-infection. When the levels of parasitemia reached 5%, mice in all groups were administered DHA via oral gavage. The intermittent preventive treatment group was administered DHA via oral gavage for only 8 days, followed by *P. berghei* ANKA infection after drug disruption.

### BrdU staining for the detection of T cells

Splenocyte proliferation was examined using flow cytometry for BrdU. The BrdU staining procedures were conducted as previously reported.^46^ For the detection of BrdU, mice in both DHA and CMC groups were administered an initial BrdU (R&D Systems, Minneapolis, Catalog No. MAB7225-SP) bolus (2 mg per 200 μL) intraperitoneally for 8 days. Gating strategy is shown in Supplementary Fig. 10.

### Immunohistochemistry experiment on Ki67 expression in splenic cells

To examine the influence of DHA on the expression Ki67 in splenic cells, paraffin-embedded and dehydrated splenic tissue sections were placed in an antigen repair buffer for antigen retrieval in a microwave oven. Next, the sections were blocked with 5% goat serum at 37 °C for 1 h and then incubated with a monoclonal anti-Ki67 antibody. The sections were then washed five times with PBS and incubated with a HRP-conjugated goat anti-mouse antibody at 37 °C for 1 h. After washing with PBS for five times, the slides were developed using a DAB substrate kit and counterstained with Hematoxylin. The results were recoded with a microscopy.

### Flow cytometric detection of spleen immune cells

Mice were euthanized with carbon dioxide and cervical dislocation. To prepare single cells, spleens were pressed through 70 µm cell strainers, and red blood cells were removed using an RBC lysis solution (BioLegend, Cat. 420301). The cells were then pre-incubated with a purified anti-mouse CD16/32 antibody (BioLegend, Cat. 101310) to block non-specific immunoglobulin binding to Fc receptors. Afterwards, the cells were incubated with specific antibodies or isotype controls, according to the manufacturer’s guidelines. A list of antibodies is available in the Supporting Information (Supplementary Table 1). The spleen immune cells were detected and analyzed using a fluorescence-activated cell sorting Aria III flow cytometer (BD Biosciences, San Jose, CA, USA); the gates were defined using the isotype and fluorescence minus one control. Gating strategy is shown in Supplementary Fig. 10.

### In vitro CD4 naïve T cell proliferation assay

Mouse lymphocytes were isolated from mouse spleens with the use of Lympholyte M (Solaibao Biological Technology Co., Ltd.) density gradient media. For obtaining naïve T cells, CD4^+^ CD62L^+^ CD44^-^ T cells were sorted using the BD FACSAria cell sorter. For in vitro experiments, DHA was dissolved in DMSO (Sigma-Aldrich). In DHA-treated-group, fresh splenic naïve T cells were incubated overnight at 37 °C in medium containing IL-2 and 0.4 μg/mL DHA. The concentration of DHA was selected based on previous study.^7^ The same amount of solvent (DMSO) and IL-2 were added to culture medium as control.

### Western blot detection of spleen immune cell proteins

Briefly, total proteins of splenic immune cells were separated by SDS-PAGE and transferred onto polyvinylidene difluoride membranes (Bio-Rad; Cat. 1620177), which were blocked with skimmed milk and incubated with primary antibodies generated from immunized rabbits overnight at 4 °C, followed by incubation with HRP-conjugated goat anti-rabbit IgG secondary antibodies (Origene; Cat. ZB-2301). A list of antibodies for Western blotting is available in the Supporting Information (Supplementary Table 1). Protein bands were quantified using ImageJ software. All experiments were repeated at least three times in triplicate for each sample.

### Proteomic and phosphoproteomic analysis of spleen cells of DHA-treated mice

Proteomic analysis was performed at the Jingjie PTM BioLab Co. Ltd. (Hangzhou, China) Proteomics Facility. The experiments were carried out according to a method described previously.^47^ Briefly, the soluble proteins from the spleen cell lysates were quantified using a BCA assay kit (Biyuntian, China) and digested in trypsin solution (at a 50:1 (m/m) protein to protease ratio) for 12 h, followed by the addition of 5 mM dithiothreitol and incubation at 56 °C for 30 min. Next, 11 mM iodoacetamide was added, and samples were incubated at room temperature for 15 min in the dark. The peptides were dispersed in mobile phase A of liquid chromatography and then separated using a NanoElute ultra-high performance liquid system (Bruker Daltonics). Phase A consisted of aqueous solutions of 0.1% formic acid and 2% acetonitrile, whereas phase B consisted of aqueous solutions of 0.1% formic acid and 100% acetonitrile. The fluid phase gradient setting was as follows: 0-70 min, 6-24% B; 70-84 min, 24–35% B; 84-87 min, 35-80% B; 87-90 min, 80% B; the flow rate was maintained at 450 nL/min. As peptides were separated by the ultra-high-performance liquid system, they contacted with the capillary ion source for ionization and the TIMS-TOF Pro mass spectrometer for analysis. We set the pulse voltage to 1.65 kV; the peptide precursors and secondary fragments were collected and analyzed via high-resolution LC/Q-TOF-MS. The scanning range of the secondary mass spectroscopy was 400–1500 m/z.

For the phosphoproteomic analysis, procedures of protein extraction and trypsinization were the same as those for proteomic analysis. Briefly, peptides were dissolved in the enrichment buffer (50% acetonitrile/6% trifluoroacetic acid). The modified peptides were eluted with 10% ammonia water, and the eluates were collected and vacuum-dried for LC/MS analysis.^48^ Parameters for fluid phase gradient settings were: 0–74 min, 6–24% B; 74–80 min, 22–35% B; 80–85 min, 35–80% B; 87–90 min, 80% B, with a flow rate maintained at 450 nL/min.

### Protein quantification and criteria for protein identification

The MS/MS data were processed using the MaxQuant v. 1.5.2.8 search engine as described.^49^ Tandem mass spectra were searched against the UniProtKB database (https://www.uniprot.org/uniprot/) Mus_musculus_10090_SP_20201214.fastaq, concatenated with a reverse decoy database. The false discovery rate (FDR) was adjusted to <1%, and the minimum score for the modified peptides was set to >40. In the first step, we changed the label-free quantitation (LFQ) intensity (*I*) level of the proteins in different samples through centralization to obtain the relative quantitative value (*R*) of the protein. The applied formula is presented in Eq. (1).

1 $${{R}}_{{{{\mathrm{ij}}}}}{{= I}}_{{{{\mathrm{ij}}}}}{{{\mathrm{/Mean}}}}\left( {{{I}}_{{{\mathrm{j}}}}} \right)$$

where i represents the sample and j represents the protein.

The proteomic and phosphoproteomic data were further normalized by centering and scaling.

### Bioinformatic analysis

Bioinformatic analysis was performed as described in our previous study, with minor modifications.^50^ Briefly, GO and KEGG functional enrichment analyses were performed using InterProScan (*p* < 0.05) and the KOG database (http://www.ncbi.nlm.nih.gov/KOG). The sequence model, with amino acids in specific modification positions, was analyzed using MoMo (motif-x algorithm).^51^

### Co-precipitation with p38 specific antibody

To investigate whether p38 directly interacts with CDK2, spleen cells from CMC and DHA-treated mice were washed five with ice-cold PBS, lysed in ice-cold Cell Lysis Buffer (Shanghai Biological Engineering Inc) containing protease and phosphatase inhibitors. Immunoprecipitation (IP) assay was performed immediately after lysis using protein A magnetic Dynabeads (Beijing Zhongyuan Ltd) and an anti-p38 antibody with five consecutive washes with IP buffer. Another IP was performed in parallel with an irrelevant control IgG. The precipitated proteins were detected with anti-P38 and anti-CDK2 antibodies, respectively, by Western blotting.

### Statistical analysis

The R program, version 4.0.3, was used for all statistical analyses. For samples in experiments that were repeatedly tested, the average value of each protein in each replicate was calculated. We then calculated the ratio of these two averages, which was applied as the final expression ratio. The two-sample two-tailed *t*-test was applied to determine the significance of the difference.

## Supplementary information

Supplementary Materials

Dataset 1

Dataset 2

## Data Availability

The datasets presented in this study can be found in online repositories. The names of the repository/repositories and accession number(s) can be found below: ProteomeXchange consortium via the PRIDE archive (accession number PXD030353, http://proteomecentral.proteomexchange.org).
