# Supplementary material for: Dihydroartemisinin regulates immune cell heterogeneity by triggering a cascade reaction of CDK and MAPK phosphorylation
Source: Signal Transduct Target Ther. 2022 Jul 11;7:222. doi: 10.1038/s41392-022-01028-5 (PMC9271464; doi:10.1038/s41392-022-01028-5)
Supplement: Supplementary file 1 — Supplementary Materials [file 41392_2022_1028_MOESM1_ESM.docx]

Supplementary Materials for

Dihydroartemisinin regulates immune cell heterogeneity by triggering a cascade reaction of CDK and MAPK phosphorylation

Qilong Li, Quan Yuan, Ning Jiang, Yiwei Zhang, Ziwei Su, Lei Lv, Xiaoyu Sang, Ran Chen, Ying Feng, Qijun Chen*

Correspondence to: qijunchen759@syau.edu.cn

**This PDF file includes:**

Supplementary Figures. S1 to S10

Supplementary Tables S1

Captions for Data S1 to S2


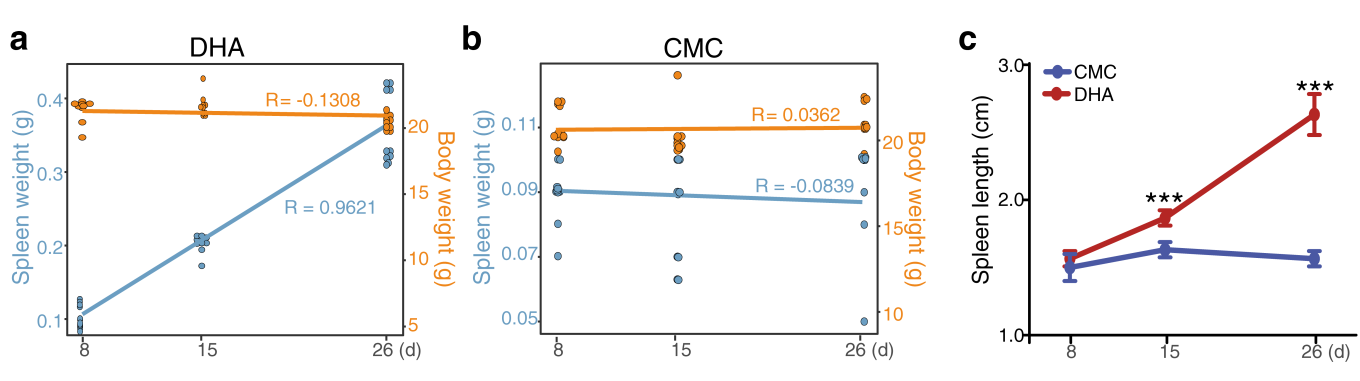
 Supplementary Figure. S1. Mice gavaged with DHA exhibited a significant increase in splenic weight relative to controls. (a and b) Correlation between spleen or body weight (g) and days after DHA or CMC treatment. (c) Splenic measurements were taken to assess maximum splenic length. Each dot represents the observed value of one mouse, and 10 mice were included in each group. Some of the values (dots) overlapped.


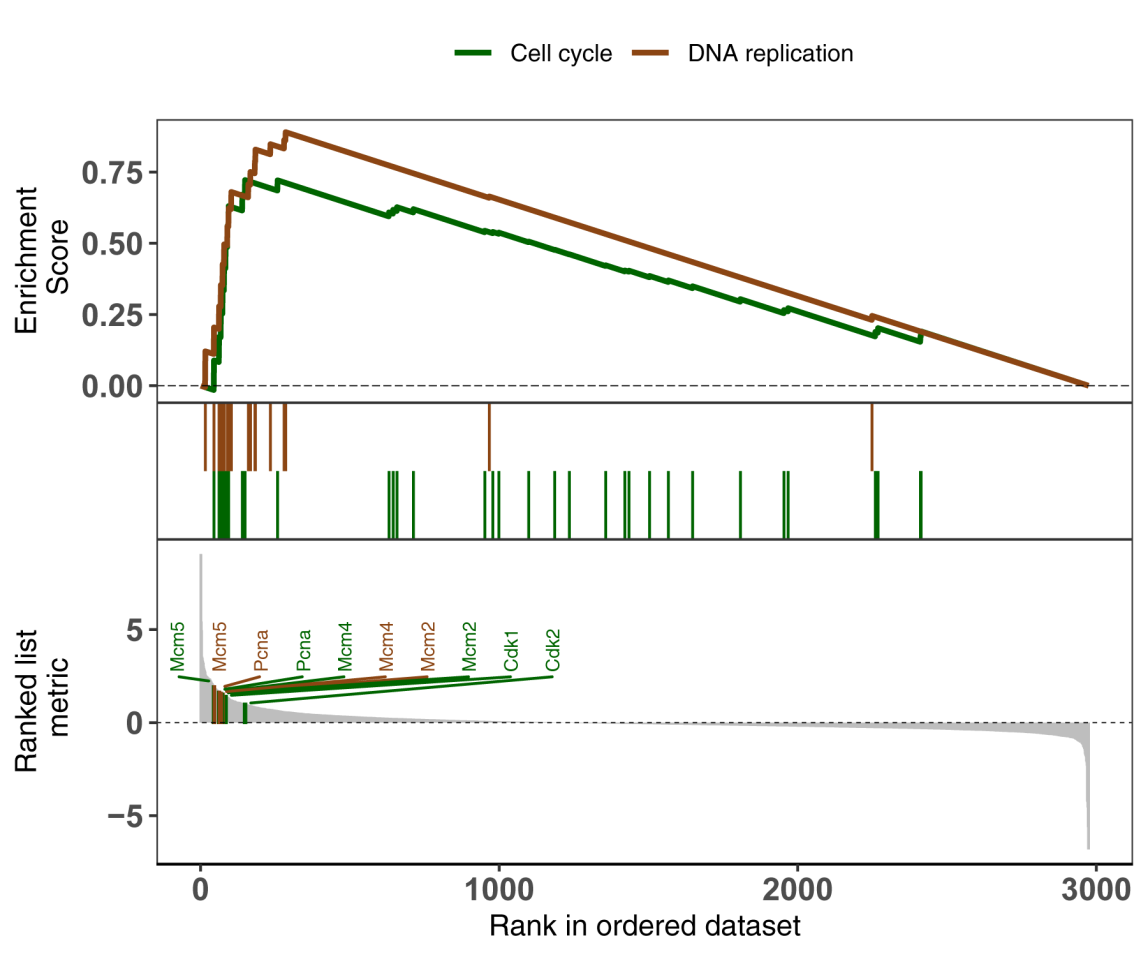


Supplementary Figure. S2. Gene set enrichment analysis (GSEA) of differentially expressed proteins between DHA and CMC treatment. GSEA revealed a highly significant enrichment for cell cycle proteins among those upregulated by DHA.


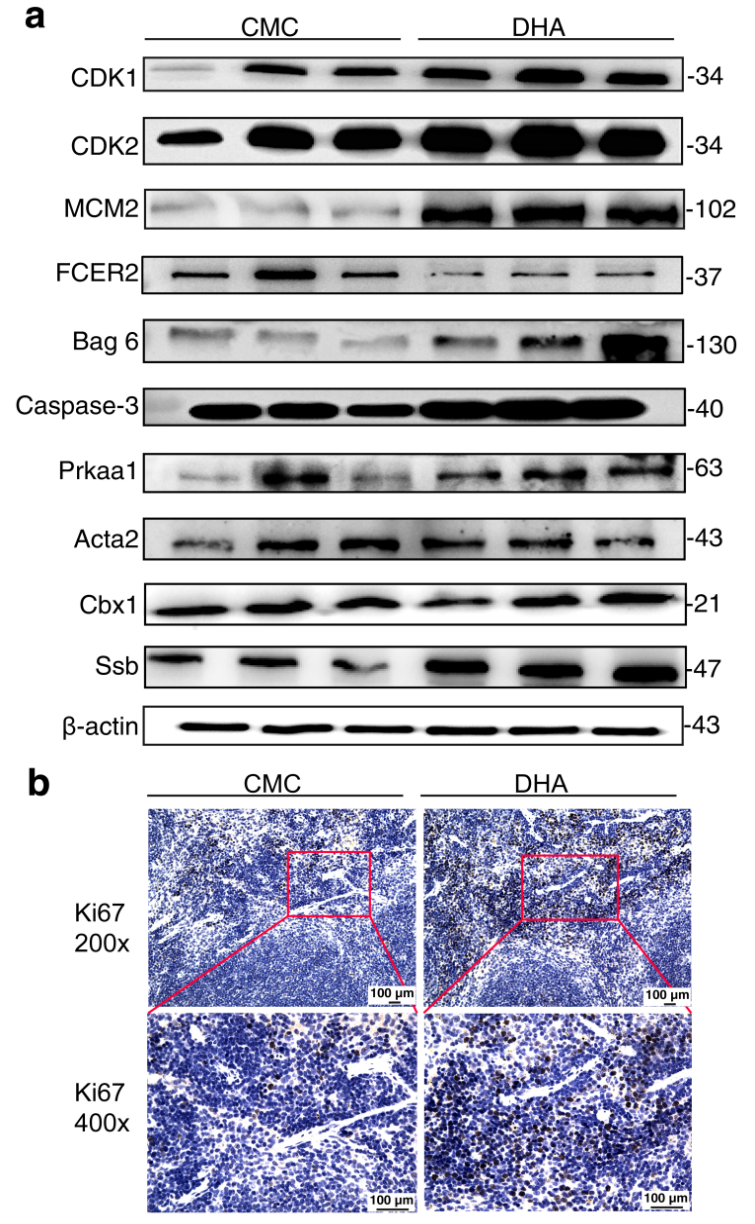


Supplementary Figure. S3. Validation of the proteomics results with Western blot and immunohistochemical staining. (a) The expression of CDK1, CDK2, MCM2, FCER2, Bag6, Caspase-3, Prkaa1, Acta2, Cbx1 and SSB in the spleens of mice treated with DHA and CMC control was compared and analyzed by Western blotting. β-actin was used as a loading control. In agreement with quantitative proteomic results, CDK1, CDK2, MCM2, Bag6, Caspase-3, Prkaa1, Ssb were upregulated whereas FCER2, Acta2 and Cbx1 were downregulated expression in DHA treated mice compare with CMC control. Molecular weight (kDa) was labeled at the right. (b) Cell proliferation of spleen samples from DHA and CMC groups was assessed by immunohistochemical staining of Ki67. DHA increased expression of the proliferation marker Ki67. Scale bar in IHC is 100 μm.


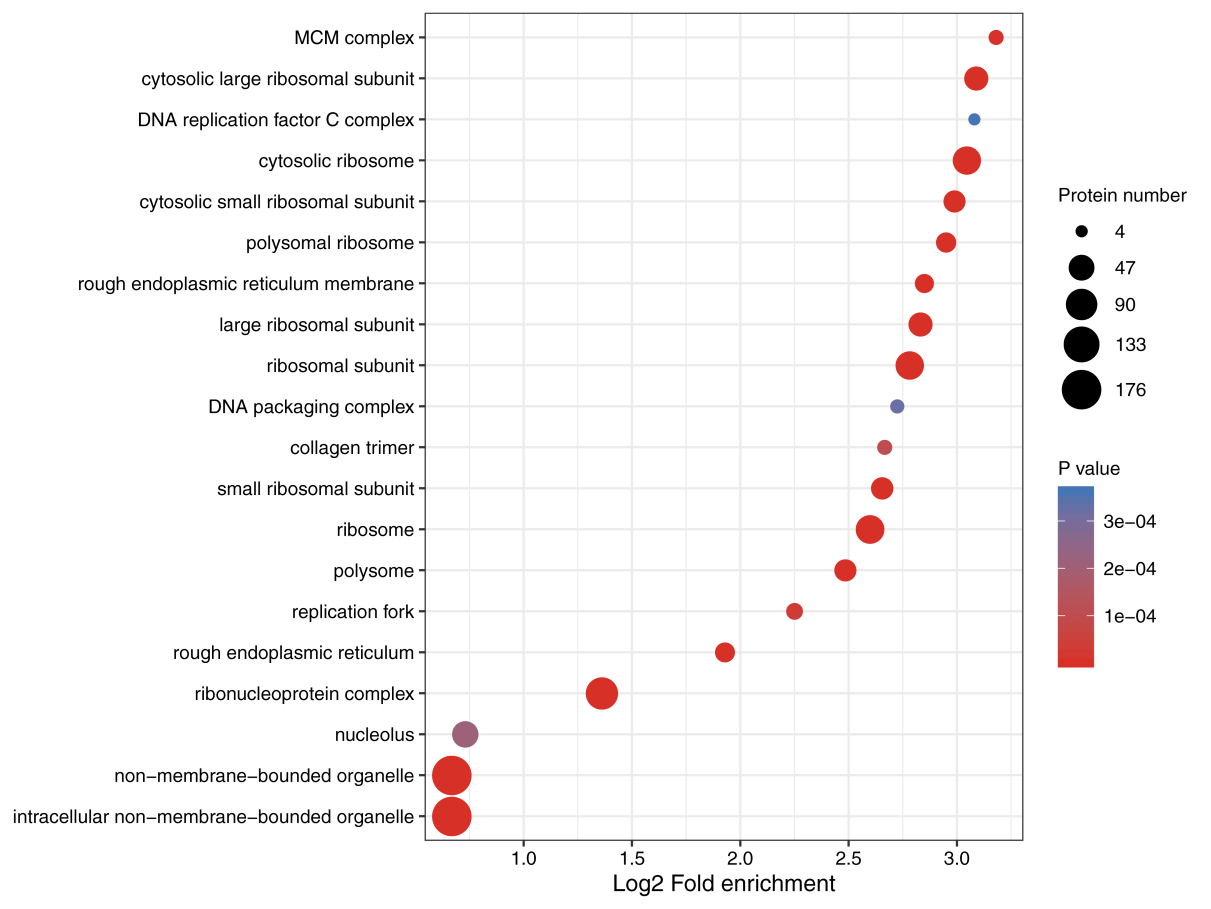


Supplementary Figure. S4. GO enrichment analysis of differentially expressed proteins between DHA and CMC treatment. GO plot showed the 20 most enriched KEGG pathways among up-regulated proteins in DHA group.


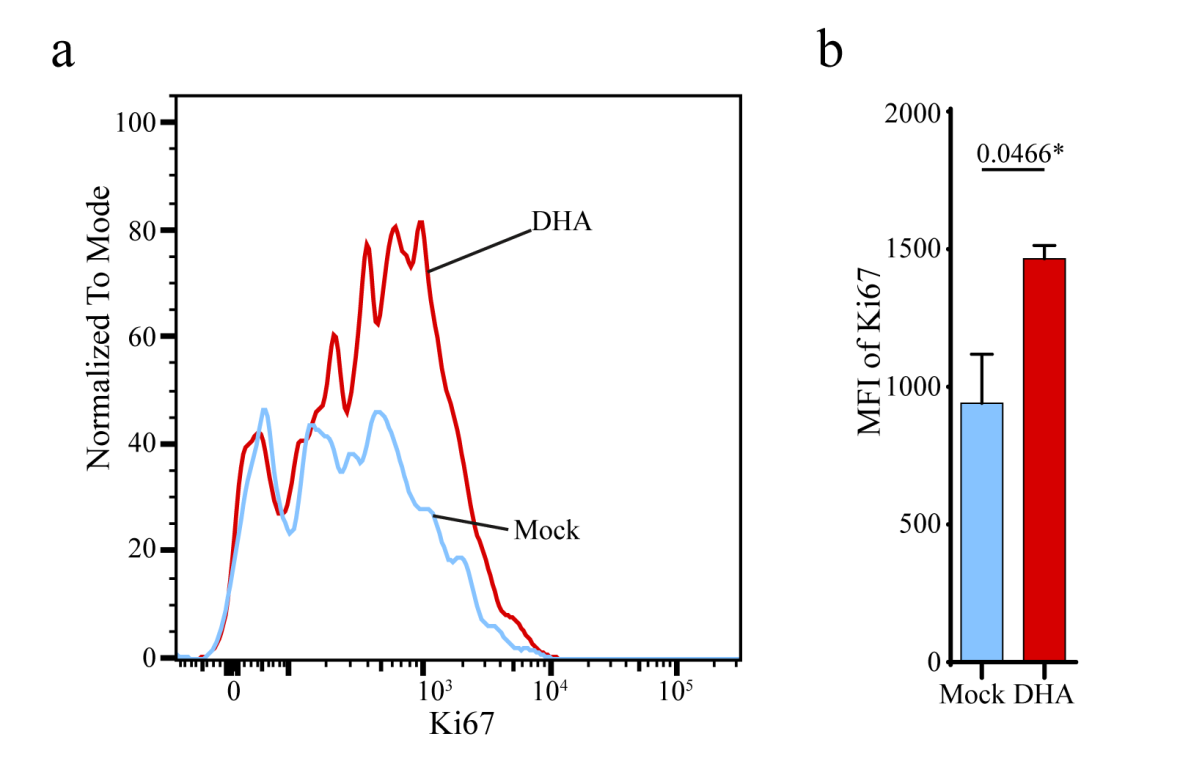


Supplementary Figure. S5. DHA upregulated expression of Ki67 in CD4 naı̈ve T-cells *in vitro*. (a) Cytometry of normalized expression of Ki67 in CD4 naı̈ve T-cells under different treatment. (b) Bar graph shows differences in Ki67 expression in CD4 naı̈ve T-cells under different treatment *in vitro*. Purified CD4 naı̈ve T cells were treated with DHA (red) and mock (blue). MFI, mean fluorescence intensity. **p* < 0.05


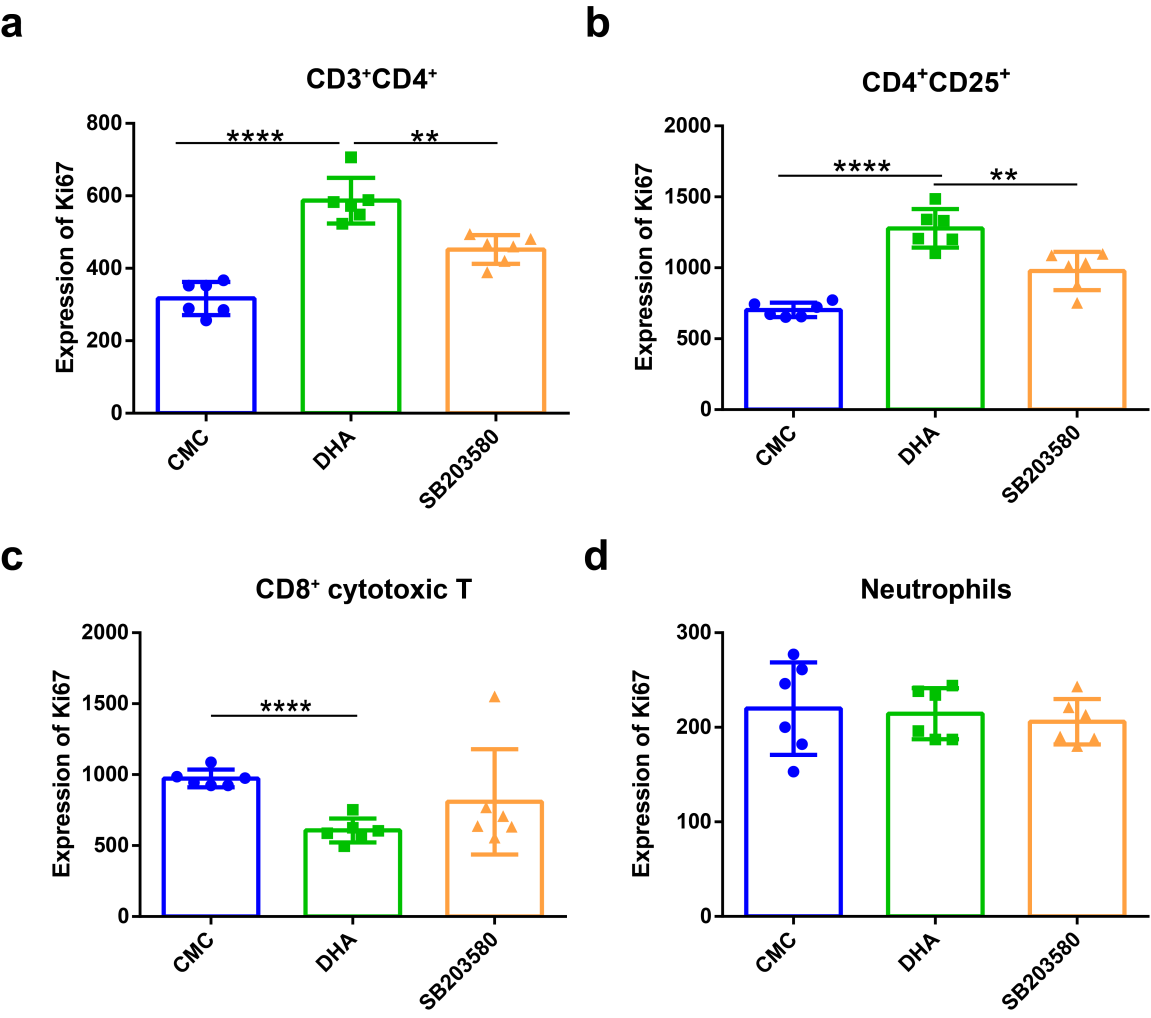


**Supplementary Figure S6. SB203580 counteracted DHA effect on Ki67 expression in T cells.** (a- d) Digits in the y axis indicate MFI levels of Ki67 expression of different cell populations of the experimental groups. The expression of ki67 on the different cell sets was calculated as MFI = positive staining (MFI)-isotype control (MFI). DHA= Dihydroartemisinin group; CMC= CMC (carboxymethyl cellulose) solvent solution control group. All mice were treated for 8 days. The DHA induced-expression of Ki67 was significantly decreased by SB203580, a specific inhibitor of mitogen-activated protein kinase (p38).

**
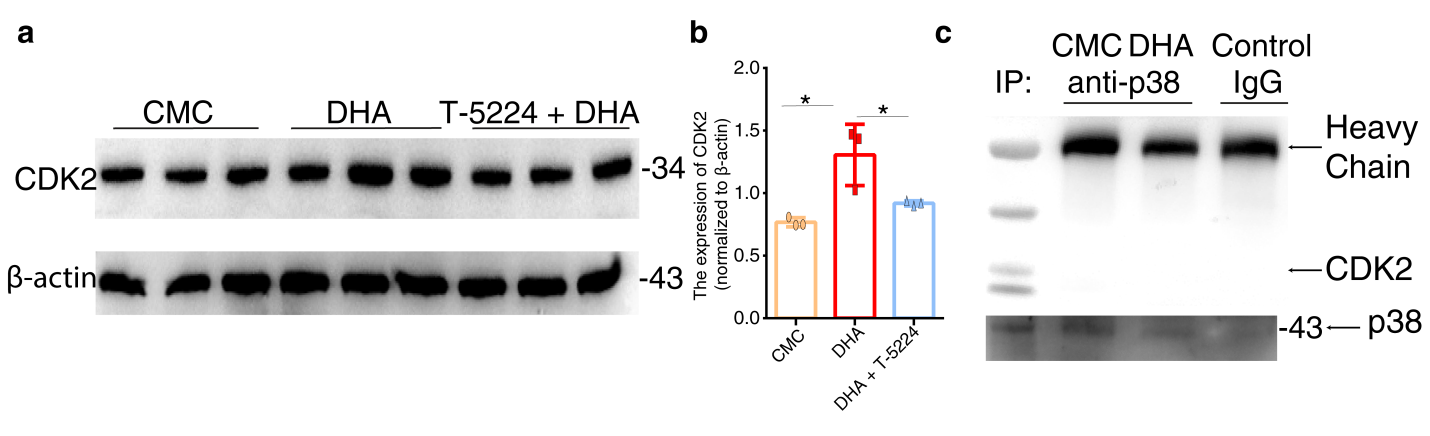
**

**Supplementary Figure S7. CDK2 was regulated by c-Fos but not by p38.** (a) The expression of CDK2 in the splenic cells of mice treated with DHA alone, DHA with the T-5224 inhibitor and CMC control was examined with specific antibodies. The upregulatory effect of DHA on CDK2 was significantly inhibited by T-5224, a specific inhibitor of C-Fos. Molecular weight (kDa) was labeled at the right. (b) Bar graph shows differences in normalized abundance of the CDK2 between CMC control, DHA treatment and DHA plus T-5224 groups. Increased CDK2 expression was observed in mice treated with DHA compared to that of CMC-treated mice, whereas CDK2 expression was greatly reduced in mice treated with T-5224 in the presence of DHA. (c) CDK2–p38 interactions were assessed using co-immunoprecipitation followed by Western blotting. The results suggested that there was no interaction of p38 with CDK2. DHA= Dihydroartemisinin group; CMC= CMC (carboxymethyl cellulose) solvent solution control group.


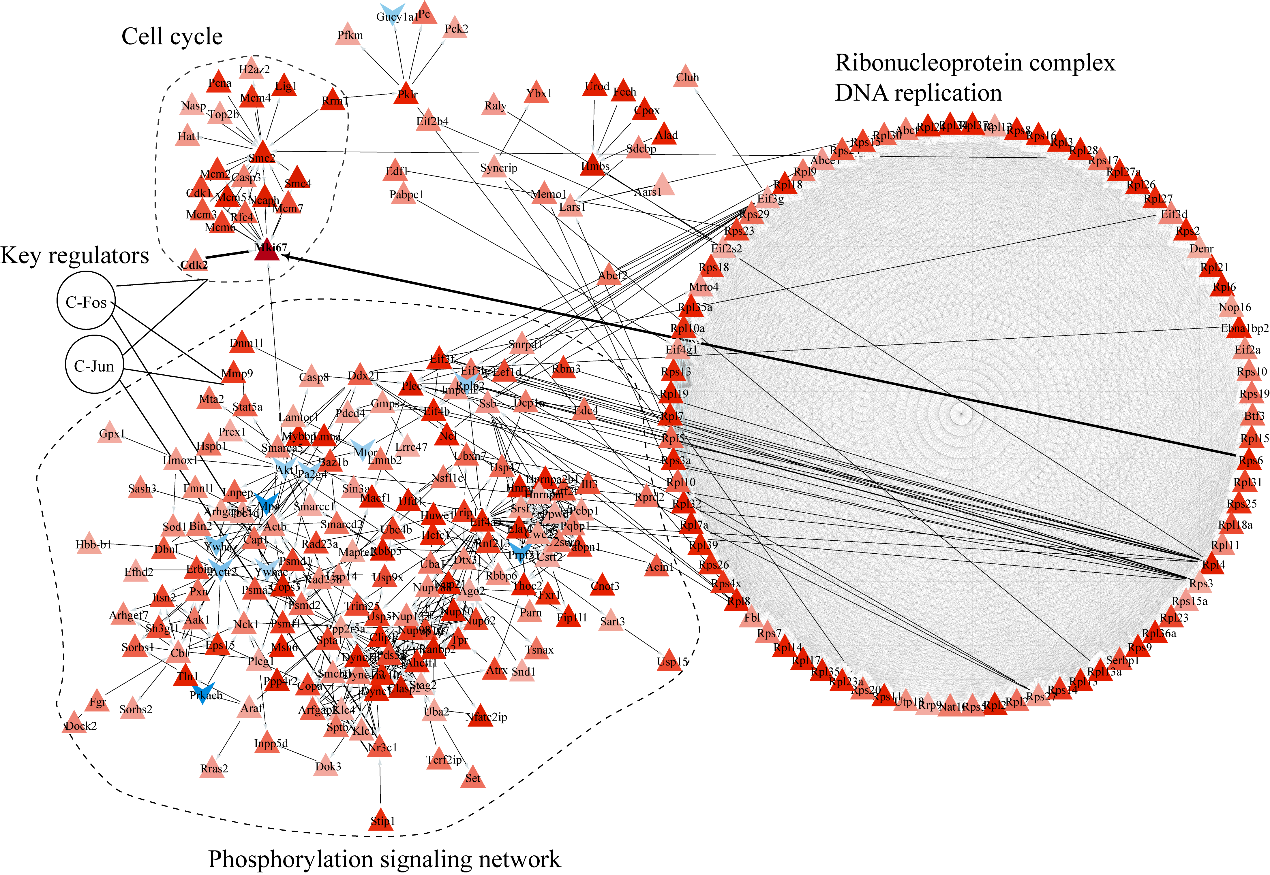


Supplementary Figure S8. Interaction networks of DHA regulated and differentially expressed proteins (DEPs). The interactive proteins were connected by lines. Markov cluster algorithm (MCL) identified 3 clusters (key regulators, phosphorylation networks and ribonucleoprotein complex) that were annotated and enriched using the GO and KEGG plugin on Cytoscape. Triangles represent DEPs, red represents upregulated proteins, and blue represent downregulated proteins in DHA-treated group compared to CMC group. Circles represent transcriptional factors. The expression abundance of the proteins was indicated by the colormetric intensity.





Supplementary Figure. S9. Intermittent preventive treatment (IPT) with DHA promoted resistance to *P.* *berghei* ANKA infection represented by parasitaemia reduction. Mice pre-treated with DHA performed resistance to *P.* *berghei* infection. **p* < 0.05; ****p* < 0.001


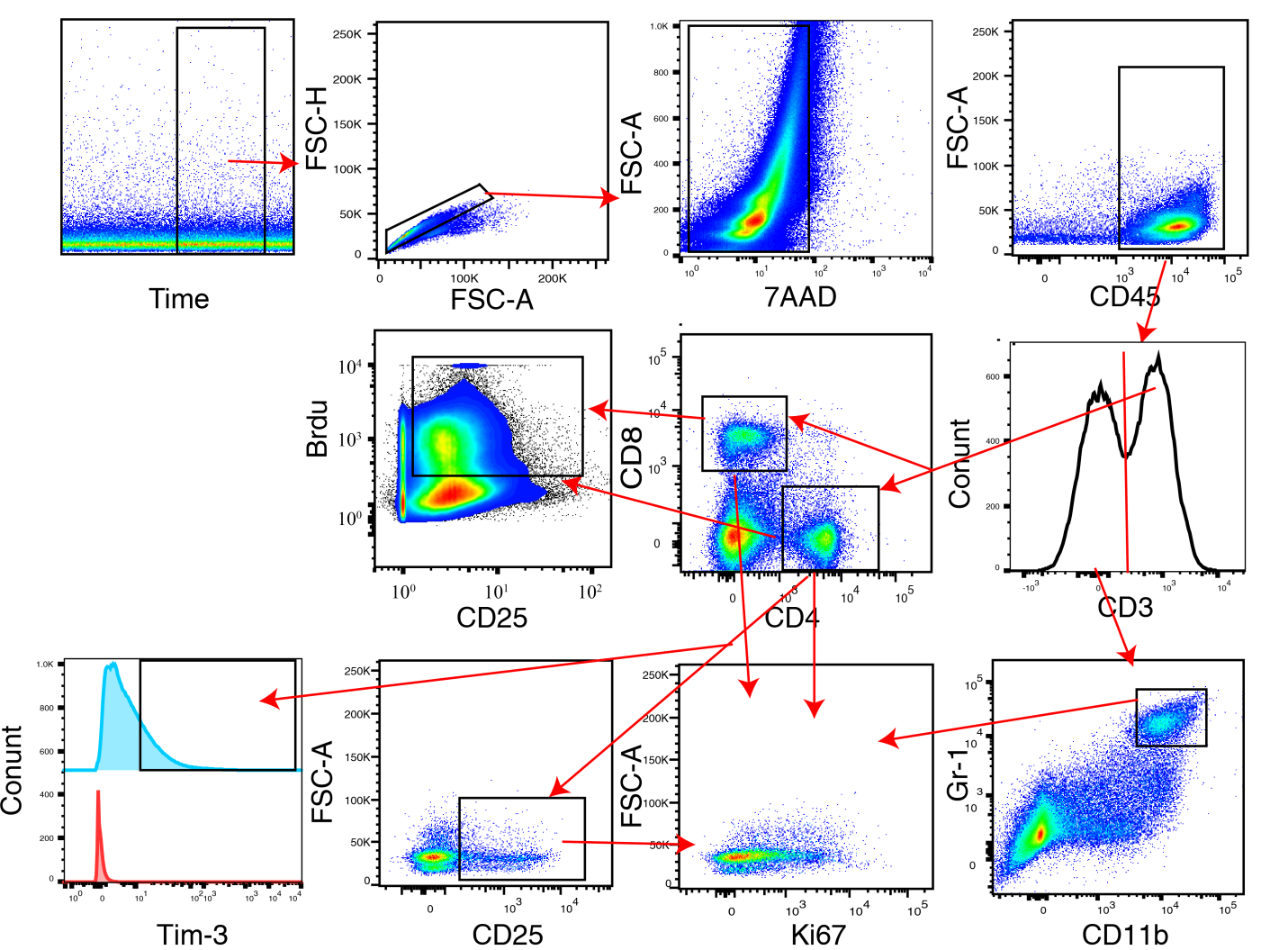


Supplementary Figure. S10. Representative gating strategy for flow cytometry.

**Supplementary Table S1. Key antibodies used in the study.**

| Reagent | Usage | | Clone or RRID | Cat.# | Source |
| --- | --- | --- | --- | --- | --- |
| Anti-phosphotyrosine rabbit pAb | | Western Blot | NA | PTM-702 | PTM BIO |
| Bromodeoxyuridine/BrdU Antibody | | Flow cytometry | NA | MAB7225-SP | RD systems |
| JNK1/2/3 Antibody | | Western Blot | AB-2835177 | AF6318 | Affinity |
| JunB Antibody | | Western Blot | AB-2835079 | AF6198 | Affinity |
| c-Jun Antibody | | Western Blot | AB-2834984 | AF6090 | Affinity |
| FosB Antibody | | Western Blot | AB-2834932 | AF5010 | Affinity |
| c-Fos Antibody | | Western Blot | AB-2833316 | AF0132 | Affinity |
| Phospho-JunB (Thr102/Thr104) | | Western Blot | AB-2845368 | AF2354 | Affinity |
| p38 MAPK Antibody | | Western Blot | AB-2835277 | AF6456 | Affinity |
| β-Actin (13E5) Rabbit mAb | | Western Blot | 13E5 | 4970 | Cell Signaling Technology |
| BAG6 Antibody | | Western Blot | NA | 8523 | Cell Signaling Technology |
| S100A8 Antibody | | Western Blot | AB-2838518 | DF6556 | Affinity |
| Fcer2 Antibody | | Western Blot | AB-2838612 | DF6650 | Affinity |
| Phospho-JNK1/2/3 (Thr183+Tyr185) Antibody | | Western Blot | AB-2834737 | AF3318 | Affinity |
| Anti-CDK2 Rabbit mAb | | Western Blot | JRMR-156 | PTM-5121 | PTM BIO |
| Anti-Ki67 Rabbit mAb | | IHC | JRMR-351 | PTM-5032 | PTM BIO |
| Phospho-p38 MAPK | | Western Blot | NA | AM063 | Beyotime Biotechnology |
| Anti-p44/42 MAPK | | Western Blot | NA | AM076 | Beyotime Biotechnology |
| Anti-Phospho-p44/42 MAPK | | Western Blot | NA | AM071 | Beyotime Biotechnology |
| Anti-CDK1 Antibody | | Western Blot | NA | ab18 | Abcam |
| Anti-MCM2 Antibody | | Western Blot | NA | ab108935 | Abcam |
| Phospho-c-Jun (Ser63) Antibody | | Western Blot | NA | AF5779 | Beyotime Biotechnology |
| Goat anti-Rabbit IgG (H+L) Highly Cross-Adsorbed Secondary Antibody | | Flow cytometry | AB-2835079 | A-11034 | Invitrogen |
| Pacific Blue anti-mouse CD45 Antibody | | Flow cytometry | 30-F11 | 103126 | Biolegend |
| APC anti-mouse/human CD11b Antibody | | Flow cytometry | M1/70 | 101212 | Biolegend |
| FITC anti-mouse Ly-6G/Ly-6C (Gr-1) Antibody | | Flow cytometry | RB6-8C5 | 108406 | Biolegend |
| PE Anti-Mouse CD3 Antibody | | Flow cytometry | 17A2 | PE-65077 | Proteintech |
| FITC anti-mouse CD3 Antibody | | Flow cytometry | 17A2 | 100203 | Biolegend |
| PE/Dazzle 594 anti-mouse CD197 (CCR7) Antibody | | Flow cytometry | 4B12 | 120122 | Biolegend |
| Alexa Fluor 647 Rat Anti-Mouse CD45RA | | Flow cytometry | 14.8 | 562763 | BD Biosciences |
| Brilliant Violet 605 anti-mouse/human CD44 Antibody | | Flow cytometry | IM7 | 103047 | Biolegend |
| PE/Cyanine7 anti-mouse CD62L Antibody | | Flow cytometry | MEL-14 | 104417 | Biolegend |

**Captions for Data S1. List of up- and downregulated proteins by DHA.**

Captions for Data S2. The list of phosphorylated proteins
